# Estimating a preference-based index for a menopause specific health quality of life questionnaire

**DOI:** 10.1186/1477-7525-3-13

**Published:** 2005-03-15

**Authors:** John E Brazier, Jennifer Roberts, Maria Platts, York F Zoellner

**Affiliations:** 1 Health Economics and Decision Science, School of Health and Related Research, The University of Sheffield, Regent Court, 30 Regent Street, Sheffield S1 4DA, UK; 2 Institute of General Practice and Primary Care, School of Health and Related Research, The University of Sheffield, Community Sciences Building, Northern General Hospital, Sheffield, UK; 3 Global Health Economics, Solvay Pharmaceuticals, PO Box 220, D-30002, Hannover, Germany

## Abstract

**Background:**

The aim of the study was to develop a menopause-specific, preference-based health-related quality-of-life (HRQoL) index reflecting both menopausal symptoms and potential side-effects of Hormone Replacement Therapy (HRT).

**Methods:**

The study had three phases: the development of a health state classification, a prospective valuation survey and the estimation of a model to interpolate HRQoL indices for all remaining health states as defined by the classification. A menopausal health state classification was developed with seven dimensions: hot flushes, aching joints/muscles, anxious/frightened feelings, breast tenderness, bleeding, vaginal dryness and undesirable androgenic signs. Each dimension contains between three and five levels and defines a total of 6,075 health states. A sample of 96 health states was selected for the valuation survey. These states were valued by a sample of 229 women aged 45 to 60, randomly selected from 6 general practice lists in Sheffield, UK. Respondents were asked to complete a time trade-off (TTO) task for nine health states, resulting in an average of 16.5 values for each health state.

**Results:**

Mean health states valued range from 0.48 to 0.98 (where 1.0 is full health and zero is for states regarded as equivalent to death). Symptoms, as described by the classification system, can be rank-ordered in terms of their impact (from high to low) on menopausal HRQoL as follows: aching joints and muscles, bleeding, breast tenderness, anxious or frightened feelings, vaginal dryness, androgenic signs. Hot flushes did not significantly contribute to model fit. The preferred model produced a mean absolute error of 0.053, but suffered from bias at both ends of the scale.

**Conclusion:**

This article presents an attempt to directly value a condition specific health state classification. The overall fit was disappointing, but the results demonstrate that menopausal symptoms are perceived by patients to have a significant impact on utility. The overall effect is modest compared to the more generic health state descriptions such as the EQ-5D. The resultant algorithm generates a preference-based index that can be used economic evaluation and that reflects the impact of this condition.

## Background

The increasing demand for economic evaluation of health care interventions has lead to a corresponding rise in the derived demand for evidence on the key parameter inputs into cost effectiveness models. One of those inputs is the health state utility value used to estimate the quality adjusted life years (QALYs) associated with an intervention. This article is concerned with estimating a preference-based measure for generating utility values for menopausal health states.

Most preference-based measures of health such as the EQ-5D, SF-6D and the Health Utilities Index 3, have a generic health state descriptive system [1-3]. However, general measures of health have been found to be inappropriate or insensitive for some medical conditions [4], and it has been found that these instruments are not sufficiently sensitive to the impact of menopausal symptoms [5,6]. There has been increasing interest in estimating preference-based indices from condition specific measures. These have often involved mapping from condition specific measures onto preference-based measures [4,7-9]. While this approach is useful, it is a second best solution for studies that did not use a generic measure and the aim is to estimate generic preference scores. However, for some conditions generic measures may not be appropriate and in this case a better solution would be to elicit preference weights for the condition specific measure [10]. There have been a number of studies published recently that have estimated conditions specific preference scores, including for Rhinitis [11], Erectile Dysfunction [12], asthma [13] and Prostate symptoms [14]. This is the first attempt to estimate a preference-based measure for menopausal symptoms.

The aim of this study is to quantify the impact of menopause-related health problems on health-related quality of life as indicated by a "strength-of-preference" index. This project had three components. The first was to construct a health state classification system for menopausal symptoms based on work by Zoellner and others [15]; secondly a sample of menopausal health states defined by the latter were then valued by means of the time trade-off; and then modelled the health state values using regression techniques to produce an algorithm for valuing all states described by the menopausal health state system.

## Methods

### The menopause-specific health state classification

A menopause-specific quality-of-life questionnaire has been developed by Zoellner and colleagues [6,15]. Initially, a pool of 39 menopause-related items – identified as being important on the grounds of two focus group sessions of peri- and postmenopausal women, literature review, and expert opinion – underwent intensive analysis to determine the degree of fulfilment of standard psychometric criteria of re-test reliability, face validity, construct validity and convergent validity.

The application of these criteria resulted in a questionnaire with 22 items grouped into 6 domains, namely (1) psychosocial, (2) physical, (3) vasomotor, (4) sexual, (5) menstrual, and (6) androgenic complaints. In order to derive a health state classification from the former, the most robust item(s) were chosen from each domain. As it was felt important to cover potential side-effects of Hormone Replacement Therapy (HRT), the menstrual domain is represented with two items – 'breast tenderness' and 'vaginal bleeding' – in the classification systems; the latter hence consists of the following seven domains of menopausal health (see table 1): hot flushes, aching joints/muscles, anxious/frightened feelings, breast tenderness, bleeding, vaginal dryness and undesirable androgenic signs. The assignment of the number as well as the descriptor of levels was performed according to the frequency distributions observed in the screening section of the postal survey (n = 785, Table 1). Each dimension contains between three and five levels and defines a total of 6,075 health states.

**Table 1:** The Menopause health state classification

**1. hot flushes**
1) You have no hot flushes
2) You get 1–3 hot flushes *per day*
3) You get 4 or more hot flushes *per day*

**2. aching joints or muscles**

1) You have no aching joints or muscles at all.
2) You have 1–3 *episodes *of aching joints or muscles *per week*.
3) You have 4 or more *episodes *of aching joints or muscles *per week*.
4) You have mild to moderate *constant *pain in your joints or muscles.
5) You have severe *constant *pain in your joints or muscles.

**3. anxious or frightened feelings**

1) You do not have anxious or frightened feelings.
2) You have anxious or frightened feelings 1–3 times *per week*.
3) You have anxious or frightened feelings 4 or more times *per week*.

**4. breast tenderness**

1) You have no breast tenderness.
2) You have *mild to moderate *breast tenderness.
3) You have *severe *breast tenderness

**5. bleeding**

1) You have no bleeding
2) You have *mild regular *(monthly) bleeding
3) You have *mild irregular *bleeding
4) You have *intense regular *(monthly) bleeding
5) You have *intense irregular *bleeding

**6. undesirable cosmetic signs (facial or body hair growth, greasy skin or acne)**

1) You have no undesirable cosmetic signs.
2) You have *mild to moderate *undesirable cosmetic signs
3) You have *severe *undesirable cosmetic signs.

**7. vaginal dryness**

1) You have no vaginal dryness.
2) You have *mild to moderate *vaginal dryness.
3) You have *severe *vaginal dryness.

### The valuation survey

The design of the survey was to elicit values for a sample of states defined by the menopausal health state classification using a variant of the Time Trade-off (TTO) on a sample of women aged 45 to 60. The key design issues were the sample of health states to be valued, the sample of respondents, the valuation technique and the interview.

### Selection of health states

It is not possible to value all the states defined by the menopausal specific health state classification. However, there is currently little guidance of the selection of states for valuation [16]. Based on past practice, the states used in this survey were selected using the orthoplan programme in SPSS. This programme generates an orthogonal array of states that need to be valued in order to estimate an additive model. This programme indicated that 49 states were necessary to estimate an additive model. It was decided to enhance these states in order to ensure some degrees of freedom and to permit some examination of interactions. Therefore, the programme selected another 47 as 'hold out' states drawn at random. This resulted in a total of 96 health states valued out of a potential of 6,075 defined by the menopausal health state classification system.

Each respondent was asked to value a sample of eight states. These states were selected from the larger sample of 96 using a stratified sampling technique to ensure that each respondent has a mix of mild, moderate and more severe states. The severity of the states has been assessed by summing the dimension levels. The states were then ranked using this sum score and divided into quartiles to identify four severity groups. Two health states have been selected at random without replacement from each severity group to form a set of eight health states. This was done another 11 times to create 12 sets of states. These 12 sets were used an equal number of times in order to ensure that each of the 96 states would be valued an equal number of times.

The original aim was to interview 150 respondents, where each respondent valued eight health states. This would mean undertaking 24 sessions with between 6–8 respondents at each session and would have resulted in 1200 observations and an average of 12.5 valuations per state.

### Selection of respondents

A previous survey was undertaken in Sheffield (UK) with the main aim of validating the new questionnaire designed to assess the health of women in mid -life. One thousand and eighty women aged 45 to 60 were randomly selected from the lists of 6 GPs in Sheffield (180 women per GP List) and sent the postal questionnaire concerning their menopausal symptoms. Of these 790 (73%) were returned. Five were dropped from further analysis due to incomplete response, so the total number was 785 responders. All responders were sent a summary of the study "Women's Health in Mid life" in December 2001 and asked if they would be interested in participating in a second phase of the study. Out of these 417 women replied saying they would like more information of the phase 2 study.

The 6 GP practices signed a consent form agreeing to these women being invited to participate in the valuation survey. Invitation letters were sent by each practice with a Patient information sheet and a Patient consent form. Out of the 417, 229 (55%) attended the interviews and completed a questionnaire.

### Valuation technique

Health states were valued using a variant of the time trade-off technique (TTO). This technique asks the respondent to choose between a fixed period of time (t) in the health state to be valued compared to a shorter period in full health (x). The amount of time spent in full health is varied until the respondent is indifferent between the two alternatives. The value of the health state is then x/t for states better than dead.

This study used a self-completed variant of TTO developed by Gudex that uses a titration procedure shown in Table 2, where the respondent is presented with two lists of values [17]. Each row has a value of 25 for t and a declining value for x, where the value of x declines by one year between each row. Twenty-five years was chosen to represent a reasonable life expectancy for this sample of respondents. The respondent is asked to indicate all the cases where they are confident they would choose A (i.e. the health state to be valued), all the cases where they would choose B (i.e. full health) and the put an equals against states where they cannot choose. There was no allowance for states worse than death, but this was felt to be an unlikely scenario for states defined by the menopausal health state classification.

**Table 2:** The time trade-off question

**Choice A**	----	**Choice B**
*25 years*		*25 years*
*25 years*		*24 years*
*25 years*		*23 years*
*25 years*		*22 years*
*25 years*		*21 years*
*25 years*		*20 years*
*25 years*		*19 years*
*25 years*		*18 years*
*25 years*		*17 years*
*25 years*		*16 years*
*25 years*		*15 years*
*25 years*		*14 years*
*25 years*		*13 years*
*25 years*		*12 years*
*25 years*		*11 years*
*25 years*		*10 years*
*25 years*		*9 years*
*25 years*		*8 years*
*25 years*		*7 years*
*25 years*		*6 years*
*25 years*		*5 years*
*25 years*		*4 years*
*25 years*		*3 years*
*25 years*		*2 years*
*25 years*		*1 year*
*25 years*		*0 years*

Please put an "**A**" against all cases where you are CONFIDENT that you would choose **Choice A**.

Please put a "**B**" against all cases where you are CONFIDENT that you would choose **Choice B**.

Please put an "**=**" against ***the case ***where you **cannot choose between Choice A and Choice B**.

### Interviews

Respondents were invited to attend interview sessions held in a room at the Institute of General Practice in Sheffield (UK). Two researchers experienced in interviewing patients coordinated these sessions. The interview began with the researchers explaining the purpose of the survey and to explain the TTO task. Patients were asked to complete an example and encouraged to ask questions in order to aid in their understanding. Once the respondents were ready, they were then asked to complete all remaining questions on their own. There were 33 such interview sessions in the survey. Respondents were reimbursed for their time and travel with a voucher for £10.

The questionnaire had questions on age, occupation, education level, general health, whether or not they had stopped menstruating and if so when, and finally whether or not they continued to take HRT. They were then asked to complete the menopausal health state classification. They then undertook a practice TTO question followed by eight TTO questions. The health states were presented in a random order to avoid the risk of an ordering effect. Finally they were asked to value their own health using a TTO question.

### Modelling

The overall aim is to construct a model for predicting health state valuations based on the menopausal health state classification. The data generated by the valuation survey described above has a complex structure, as they are skewed and health state valuations are clustered by respondent. Disentangling the respondent effect is a complex task and can only be tackled at the individual level, where each valuation is regarded as a separate observation, rather than using the mean value for each health state. The former has the advantage of greatly increasing the number of degrees of freedom available for the analysis (from 96 to over 1200) and enabling the analysis of respondent background characteristics on health state valuations.

A number of alternative models have been proposed for estimating preference functions from health data [12,14,15]. The general model has been defined elsewhere as [12]:

*y*_*ij *_= *g *(*β*'**x**_*ij *_+ *θ*'**r**_*ij *_+ *δ*'**z**_*j*_) + *ε*_*ij *_    (1)

where *i *= 1, 2, ..., n represents individual health state values and *j *= 1,2, ..., m represents respondents. The dependent variable, *y*_*ij*_, is the TTO score for health state *i *valued by respondent *j*. **x **is a vector of binary dummy variables (x_*δλ*_) for each level *λ *of dimension *δ *of the classification. Level *λ *= 1 acts as a baseline for each dimension, so in a simple linear model, the intercept represents state 1111111, and summing the coefficients of the 'on' dummies derives the value of all other states.

The **r **term is a vector of terms to account for interactions between the levels of different attributes. **z **is a vector of personal characteristics that may also affect the value an individual gives to a health state, for example, age, sex and education. The role of personal characteristics is not discussed in this paper. *g *is a function specifying the appropriate functional form. *ε*_*ij *_is an error term whose autocorrelation structure and distributional properties depend on the assumptions underlying the particular model used.

This is an additive model, which imposes no further restrictions on the relationship between dimension levels of the classification. For example, it does not enforce an interval scale between the levels of each dimension and does not impose ordinality on the levels.

OLS assumes a standard zero mean, constant variance error structure, with independent error terms, that is cov(*ε*_*ij*_*ε*_*i*'*j*_) = 0, *i*≠*i*'. This specification ignores the clustering in the data and assumes that each individual health state value is an independent observation, regardless of whether or not it was valued by the same respondent. An improved specification, which takes account of variation both within and between respondents, is the one-way error components random effects model. This model explicitly recognises that n observations on m individuals is not the same as n × m observations on different individuals. Estimation is via generalised least squares (GLS) or maximum likelihood (MLE).

Analysis of first order interactions alone is problematic, since the large number of possible interactions means there is a risk of finding some are significant purely by chance. We have therefore adopted the approach used in other studies of using summary terms for describing interactions [16,18]. Extreme level dummies were created to represent the number of times a health state contains dimensions at the extreme ends of the scale [18]. Least severe is defined as level 1 on each dimension. Most severe is defined as the bottom level of each dimension. These are used to create dummy variables LEAST and MOST which take a value of 1 if any dimension in the health state is at the least (most) severe level, and 0 otherwise.

Finally we consider alternative functional forms – *g *in (1) – to account for the skewed distribution of health state valuations. Four functional forms are used. Firstly, a Logit transformation and two complementary log-log transformations suggested by Abdalla and Russell. [19] These are chosen to map the data from the range (-1,1) to the range (-∞,∞) via the unit range (0,1). Secondly, a Tobit transformation which, although designed to deal with truncated data, can approximate for the left skew in this data, where 25% of the values lie between 0.9 and 1. Specifying a Tobit model with upper censoring at 1 does this.

All modelling will be done using STATA 7.0 and SPSSWin.

## Results

### Respondents

The characteristics of the 229 interviewed women are presented on Table 3. Their mean age was 54 with a range between 46 and 61. Seventy four percent had stopped menstruating and the average time to since they last menstruated was 64 months. A third had taken HRT in the last month. The respondents reported their general health to be in the mid-range of the excellent to poor scale. The seven menopausal symptoms were highly prevalent, with two thirds experiencing aching joints and muscles, nearly half reporting hot flushes and vaginal dryness and around one third experiencing anxiety or fright, breast tenderness and cosmetic signs. The mean valuation of their current health state by the TTO was 22.8 (SD = 4.4) which translated into a health state utility value of 0.91.

**Table 3:** Characteristics of respondents

	**Full sample n = 229**
Age: mean (s.d)	53 (SD)
Highest qualification	%
Degree	26
A levels	11
Other	63
Self-rated general health:	%
Excellent	7
Very good	44
Good	31
Fair	14
Poor	4
***Reporting the following:***	%
Hot flushes	45
Aching joints or muscles	74
Anxious or frightened	37
Breast tenderness	31
Bleeding	23
Cosmetic signs	35
Vaginal dryness	45
TTO own valuation	22.8 (4.4)
Stopped menstration	74
Average time since stopped menstruation (months)	64 (72)
Taken HRT in last month	35

Thirty respondents were excluded from the modelling data set, leaving 199. Respondents were excluded due to ambiguity in the responses to the (self-administered) questionnaire. The main sources of ambiguity were the mixing up of responses (e.g. ticks and crosses appearing the wrong way around) and large gaps between the responses with no indication of the appropriate point of indifference. There were also a number of individual responses elicited from 199 respondents that had to be excluded due to similar ambiguities. These exclusions left 1580 health state values across the 96 health states for modelling, a final completion rate of 86% of all questions asked at interview.

### Health state values

**Figure 1:**
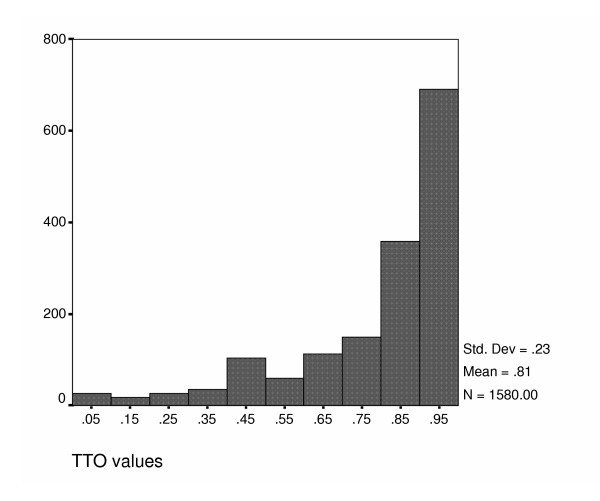
Histogram for TTO values.

Descriptive statistics for 50 of the 96 states are presented on Table 4. Each health state is valued on average 16.5 times, which exceeds the original target. Mean health state values range from 0.48 to 0.98 with large standard deviations. The median values usually exceed the mean values, reflecting the highly skewed nature of the data. This skewness is even more apparent at the individual level, as shown in the histogram presented in Figure 1. Very few health states values were 1.0 (32/1580) indicating that that most respondents were willing to trade time for quality of life, however 31% were at the next possible value of 24.5 years. At the other end of the scale, only three had a value of zero where it might have been possible that respondents regarded these states as worse than death.

**Table 4:** Descriptive Statistics for 50 health state valuations

**State**	**Mean**	**n**	**s.d.**	**Median**	**maximum**	**Minimum**
1112311	0.93	16	0.08	0.98	0.98	0.78
1112422	0.87	16	0.16	0.90	1.00	0.38
1112433	0.79	15	0.24	0.94	0.98	0.26
1113122	0.88	18	0.16	0.94	0.98	0.42
1113232	0.79	17	0.23	0.82	1.00	0.02
1113512	0.82	15	0.21	0.94	0.98	0.38
1113531	0.80	17	0.22	0.90	0.98	0.38
1121223	0.83	18	0.16	0.88	0.98	0.42
1121331	0.86	16	0.17	0.90	1.00	0.42
1211522	0.87	15	0.19	0.98	1.00	0.38
1322231	0.71	18	0.31	0.84	0.98	0.00
1323123	0.81	14	0.22	0.90	0.98	0.22
1323231	0.72	18	0.27	0.84	0.98	0.22
1331412	0.80	15	0.23	0.90	0.98	0.22
1331432	0.56	13	0.22	0.54	0.94	0.06
1332213	0.77	18	0.23	0.88	0.98	0.26
1333112	0.84	18	0.19	0.92	0.98	0.42
1413211	0.68	13	0.22	0.74	0.90	0.10
1413513	0.78	15	0.22	0.82	0.98	0.22
1423133	0.76	18	0.21	0.80	0.98	0.26
2221511	0.67	13	0.25	0.74	0.98	0.06
2223131	0.83	17	0.14	0.82	1.00	0.54
2231312	0.89	15	0.13	0.98	1.00	0.54
2233333	0.48	13	0.28	0.42	0.98	0.06
2311511	0.87	18	0.16	0.92	1.00	0.42
2313123	0.96	16	0.04	0.98	1.00	0.86
2321122	0.70	13	0.24	0.74	0.98	0.10
2332413	0.54	13	0.26	0.54	0.98	0.06
2421212	0.92	17	0.09	0.98	1.00	0.70
2421322	0.82	18	0.19	0.92	0.98	0.42
2521323	0.65	18	0.30	0.78	0.94	0.00
2523423	0.71	16	0.23	0.74	0.98	0.34
2532323	0.83	18	0.24	0.88	0.98	0.00
2532531	0.71	16	0.26	0.72	0.98	0.14
2533311	0.77	15	0.31	0.94	1.00	0.04
3121111	0.92	17	0.15	0.98	1.00	0.42
3121533	0.81	18	0.20	0.86	0.98	0.34
3132211	0.83	16	0.19	0.90	1.00	0.34
3133412	0.79	17	0.21	0.90	0.98	0.34
3133521	0.67	15	0.26	0.78	0.98	0.14
3232433	0.77	15	0.27	0.90	0.98	0.18
3311433	0.75	17	0.24	0.82	0.98	0.42
3312321	0.89	16	0.14	0.94	0.98	0.46
3412222	0.80	18	0.19	0.86	0.98	0.42
3413111	0.83	18	0.29	0.94	0.98	0.06
3422113	0.82	16	0.14	0.82	0.98	0.56
3422412	0.78	15	0.29	0.90	1.00	0.06
3431133	0.80	16	0.19	0.86	0.98	0.38
3433532	0.71	18	0.32	0.80	0.98	0.02
3511211	0.80	17	0.23	0.86	0.98	0.38

Please put an "**A**" against all cases where you are CONFIDENT that you would choose **Choice A**. Please put a "**B**" against all cases where you are CONFIDENT that you would choose **Choice B**. Please put an "**=**" against ***the case ***where you **cannot choosebetween Choice A and Choice B**.

### Modelling

#### Basic models: main effects

The Breusch-Pagan test for individual effects suggests these are important (*χ*^2 ^= 25585.15, P = 0.000) and Hausman's test suggests random rather than fixed effects is the appropriate specification (*χ*^2 ^= 27.11, P = .035), therefore only Random Effects (RE) and mean models are presented in Table 5. The main effects dummies in each model represent levels of each dimension of the menopausal health state classification. These are expected to have a negative sign and to increase in absolute value within each dimension. It would be inconsistent with the scale for the absolute value to decrease when moving to a worst level within a dimension.

**Table 5:** Models

	**Main effects only**		**Interaction effects**	
	**(1)**	**(2)**	**(3)**	**(4)**
	**RE**	**Mean**	**RE**	**Mean**
c	**0.912**	**0.917**	**0.925**	**0.879**
HF2	0.007	-0.008	0.006	-0.005
HF3	-0.006	0.008	-0.002	0.013
AJ2	-0.016	-0.013	-0.016	-0.0106
AJ3	**-0.026**	**-0.062**	**-0.024**	-0.062
AJ4	**-0.023**	-0.022	**-0.022**	**-0.021**
AJ5	**-0.070**	**-0.085**	**-0.066**	-0.08
EM2	-0.012	-0.018	-0.012	**-0.018**
EM3	**-0.034**	**-0.057**	**-0.029**	-0.051
BT2	**-0.018**	-0.002	**-0.018**	0.000
BT3	**-0.033**	**-0.039**	**-0.028**	-0.032
BL2	**-0.041**	-0.026	**-0.039**	**-0.024**
BL3	**-0.057**	-0.025	**-0.057**	-0.022
BL4	**-0.066**	**-0.058**	**-0.068**	-0.054
BL5	**-0.062**	-0.043	**-0.059**	**-0.037**
COS2	-0.004	0.010	-0.003	0.014
COS3	**-0.015**	-0.028	**-0.011**	-0.024
VAG2	0.006	-0.008	0.006	-0.006
VAG3	**-0.024**	**-0.035**	**-0.02**	-0.029
MOST			**-0.026**	**-0.013**
LEAST			0.002	**0.035**
N	1580	96	1580	96
adj R^2^	0.040	0.178	0.039	0.164
inconsistencies	3	2	2	3
MAE	0.056	0.053	0.065	0.0552
No > |0.05|	37	36	47	36
No > |0.10|	14	15	17	15
t(mean = 0)	-0.334	†	-0.344	†
JBPRED	**34.789**	**20.587**	**36.089**	**17.028**
LB	**214.99**	**124.92**	**218.04**	**150.44**

Estimates shown in **bold **are significant at t_0.10; _† Mean error is zero by definition.

In the RE model (1), the coefficients have the expected negative sign for all main effect dummies except HF2 and VAG2, but neither of these is significant. There are 13 significant coefficients, including the constant term. There are three inconsistencies involving significant coefficients, AJ3 to AJ4, BL2 to BL3 and BL4 to BL5. The mean model has a better explanatory power than the OLS model (not shown), but has only seven significant coefficients that produce just two inconsistencies.

The ability of the mean and RE main effects models to predict health state valuations within the data set is presented at the bottom of Table 5. The main effects models have similar mean absolute errors, though it is slightly lower for the mean model. The proportion of errors greater than 0.05 and 0.1 is also very similar at 39% and 15% respectively. The JB test found evidence for non-normality of errors for both the models.

An important problem has been identified by the Ljung-Box statistics that reveal significant autocorrelation in the prediction of all the models. Plots of actual against predicted errors reflect a tendency to over predict at the lower end and under predict at the upper end. The model was re-estimated using a Tobit procedure, but this did not improve the predictive performance of the model. Applications of the logit and complementary log functions also did not improve model performance.

### Interactions

The RE and mean models in Table 5 include dummy variables for MOST and LEAST, which take the value of 1 if any dimension is at the most or least severe level respectively. The coefficients associated with these dummies suggest a further negative impact when any dimension is at its worst level and a slight positive impact from having any dimension at the least severe level. These coefficients were significant for some models. However, the coefficients on the main effects have been slightly reduced by these additional dummies, particularly the worst levels of AJ5 and BL5. Furthermore, the addition of these variables has not significantly improved either model

## Discussion

The paper presents the results of a study aiming to estimate preference functions for the menopausal health state classification. The preference models look credible in terms of the coefficients, though there are a number of problems with the models predictive performance. This paper supports the findings of other studies, that it is the feasible to estimate condition specific preference-based indices [11-14].

It was perhaps more ambitious than other published studies in that it attempted to estimate values for a large health state classification, where it was not possible to directly value all states. It was the first study using statistical inference to model health state values. The explanatory power of the models is not high. This is due to the high variability around the health state means, which may have been a result of the self-completed format of the TTO task. It may also have been due to comparatively low number of observations per state.

The classification describes health states that are mild compared to the full range of states described by descriptive systems such as the EQ-5D or HUI3 that reflects the nature of the conditions. The specific values found for our instrument have tended to be more skewed at the upper end than the generic measures, such as the EQ-5D. This was also found for a number of other conditions, with the lowest value for Erectile Dysfunction being 0.74 [12] and 0.87 for prostate symptoms [14]. However this seems to be a consequence of the comparatively mild impact of this condition, because a preference scale for Asthma had a lowest value of 0.04 [13] and 0.15 for Rhinitis [11]. For milder conditions a valuation technique such as TTO that relies on trading quality with survival may be rather insensitive for some respondents, which is reflected in the higher proportion of people indicting the first response choice down the scale. This might suggest that more effort needs to be made to develop variants of the TTO and SG that allow milder states to be valued with sufficient sensitivity. One approach would be the use of chaining, where each mild state is valued against full health and a lower anchor that is better than being dead, which in turn has been valued against full health [20]. However, this has been shown to produce biased estimates [21,22].

A further problem may have arisen from the descriptive system. The 2 or 3 inconsistencies between coefficients may be due to possible ambiguities in the health state classification. The ranking of AJ3 and AJ4 is ambiguous, since it is possible for 4 or more episodes per week of pain to be worse than mild to moderate constant pain. Also for BL, some people may regard irregular bleeding as better than regular bleeding. Such differences of opinion in the population in the ranking ordering of some levels would reduce the fit of the models.

Of more concern is the evidence for systematic patterns in the residuals resulting in over prediction at the lower end and under predicting at the upper end of the range. The MVH group was able to solve this problem in their valuation of the EQ-5D by the inclusion of an interaction term. The inclusion of interaction terms in this study had little impact on the problem. The application of various transformations to the dependent variable also did not solve this problem.

The models nonetheless provide a basis for valuing menopausal health states using this health state classification. The coefficients are consistent with the cordiality of the health state classification and the size of the mean absolute error of 0.055 to 0.065 is comparable to that achieved in other models [16]. The addition of interaction terms did not improve the model and tended to offset the main effects, therefore it is not proposed to recommend the models with interactions. The choice of models is between the random effects and mean main effects models (i.e. (1) and (2)). Given the mean model is slightly better in terms of fit and numbers of inconsistencies this is the one recommended for use.

The estimation of preference weights for condition specific quality of life has been questioned by some health economists as to its value [23]. The argument for using condition specific descriptive systems is that they are likely to be more sensitive to changes in the condition than generic measures and more relevant to the concerns of patients. On the other hand, condition specific measures often focus on symptoms and it could be argued this concentrates the mind of the respondent on the negative aspects of the conditions. This may have a framing effect that produces lower values because the respondents are not thinking about other aspects of their lives unaffected by the condition. However, the risk of this was reduced by selecting women in the age range of 45–60, most of whom had experienced menopausal symptoms and would have a realistic view of the likely impact of the condition.

The argument for using condition specific descriptive systems is that they are going to better reflect the impact of the condition on a patient's life. However, provided the descriptive system is valued on the same full health – death scale using the same variant of the same valuation technique using a comparable population sample, then the valuations should be comparable. Any remaining differences in values should be a legitimate consequence of the descriptive system. However, this assumes that the value of a dimension is independent of those dimensions outside of the descriptive system and this requires empirical testing. Despite these arguments, there has been increasing interest in estimating condition specific preference measures of health because the analyst often only has condition specific data and wishes to use them to undertake an economic evaluation, or the analyst feels a generic measure is not appropriate for the condition.

## Conclusion

The advantages of using a condition specific descriptive system over a generic are that it should be more sensitive to improvements in health. However, the overall fit was disappointing. The results demonstrate that menopausal symptoms are perceived by patients to have a significant impact on utility, but the overall effect is modest compared to the more generic health state descriptions such as the EQ-5D. This research has also demonstrated the problems that can be encountered when trying to value a comparatively mild condition. The resultant algorithm generates a preference-based index that can be used economic evaluation and that reflects the impact of this condition.

## Authors' contributions

JB led the project, including the design of the valuation study and undertaking much of the analysis. MP contributed to the design of the valuation survey and undertook the interviews. JR provided a key input into the econometric analyses. YZ designed the menopausal health state classification and contributed to the overall design of the study. All authors contributed to the writing of the paper.
